# Challenging behaviours: Views and preferences of people with intellectual disabilities

**DOI:** 10.1111/jar.12631

**Published:** 2019-06-11

**Authors:** Ria Wolkorte, Ingrid van Houwelingen, Marieke Kroezen

**Affiliations:** ^1^ Intellectual Disability Medicine Department of General Practice Erasmus MC University Medical Center Rotterdam The Netherlands; ^2^ Research group Knowledge analysis societal security HU University of Applied Sciences Utrecht The Netherlands

**Keywords:** challenging behaviour, diagnosis, ID, interventions, medication, views

## Abstract

**Background:**

Challenging behaviour is a common problem among people with ID and in services for people with ID. This paper aims to provide an overview of the views and preferences of people with ID on challenging behaviour.

**Method:**

Semi‐structured interviews were conducted with thirteen adults with mild to moderate ID and seven proxies (family or close associates of adults with ID who were unable to communicate and/or with severe or profound ID) in the Netherlands. The interviews were audio‐recorded, transcribed and analysed thematically.

**Results:**

People with ID and (a history of) challenging behaviour have clear views and preferences on factors related to challenging behaviour, assessments, non‐pharmacological and pharmacological interventions and health professionals’ approach.

**Conclusions:**

The identified views and preferences of people with ID are not always in accordance with current procedures and treatments for challenging behaviour and should be included in future care processes and research.

## 1 INTRODUCTION

Challenging behaviour is a common problem among people with ID and in services for people with ID (Bowring, Totsika, Hastings, Toogood, & Griffith, 2017; Emerson et al., 2001; De Winter, Jansen, & Evenhuis, 2011). It is a frequent reason for referral to specialist intellectual disability services (Pickard & Akinsola, 2010) and the primary reason for referral to specialist clinics of physicians for people with ID in the Netherlands (NVAVG, 2016). Likewise, within general practice, behavioural and psychiatric problems are the most prevalent problems among patients with ID (Bekkema, De Veer, & Francke, 2014). While the term “challenging behaviour” has been much debated, it has come to replace a number of related terms including abnormal, aberrant, disordered, disturbed, dysfunctional and maladaptive behaviours, and gradually became the accepted term by many professionals (Emerson & Einfeld, 2011). The term describes a wide range of behaviours including aggression, property destruction, self‐injury and stereotypy (Lloyd & Kennedy, 2014). In this paper, the present authors build upon the definition of challenging behaviour by Emerson & Einfeld (Emerson & Einfeld, 2011) and expand it further by including internalizing behaviour, such as depressive or anxious behaviour. The authors acknowledge that the inclusion of internalizing behaviour is not standard in international definitions. However, the reason to include internalizing behaviour is to align with the definition of the Dutch multidisciplinary guideline on challenging behaviour. Hence, for the purpose of this article, challenging behaviour is defined as: “internalizing and/or externalizing behaviour(s) that is perceived to be socially or culturally undesirable in a specific context by the person and/or the environment, and of such an intensity, frequency or duration that it is detrimental, stressful or harmful for the person and/or the social environment.” This definition emphasizes the interaction between the person and his or her context in the development and persistence of challenging behaviour.

While challenging behaviour can take on many forms, one common characteristic of all these behaviours is their negative effect. Challenging behaviour has a range of negative impacts, including a lower quality of life for the person concerned, a negative impact on the emotional well‐being of family members, staff and fellow residents, reduced access to community services and (avoidable) high consumption of specialist, and therefore costly, services (Griffith, Hutchinson, & Hastings, 2013). As a result, challenging behaviour has received substantial attention in the literature, especially its epidemiology, risk factors and intervention research (Griffith et al., 2013).

It is important to note that challenging behaviour is not a diagnosable disorder. It should be considered as a means of communication or symptom of an underlying problem, often serving a function for the person with ID. The consensus among healthcare professionals is that interventions should preferably not be focused on reducing the symptoms, but rather focus on the person, behaviour and context through a multicomponent intervention (Gore et al., 2013). Interventions could for example be targeted at (a combination of) physical or mental health, personal skills or physical or social environmental factors, to improve quality of life and reduce challenging behaviour.

In view of the ongoing shift in thinking about the care for people with ID, which puts increased emphasis on self‐determination, equal participation and autonomous choices for people with ID (Stancliffe, 2001; Wehmeyer, 2005), one would expect that the views of people with ID and challenging behaviour also received considerable attention in the scientific literature. However, as Griffith et al. (Griffith et al., 2013) pointed out, this is not the case. While their review on the experiences of individuals with ID and challenging behaviour in relation to received service supports and interventions is important in this regard (Griffith et al., 2013), their overview also draws attention to a number of limitations in the current research. Most notably, the studies included in the review by Griffith et al. were conducted in only three countries (UK, USA and Canada), with 14 of the 17 originating from the UK. Their review also revealed a paucity of information about service user experiences of specific psychological interventions designed to help manage their challenging behaviour. In view of the prominence of challenging behaviour in the care for people with intellectual disabilities, its large negative impact and the importance of incorporating the experiences of people with ID in research and policy, this paper focuses on the views and preferences of people with ID on various aspects of challenging behaviour and related care processes in the Netherlands.

The aim of this paper was to provide an overview on the views and preferences of people with ID on various aspects of challenging behaviour. The following research questions are addressed. What are the views (and preferences) of people with ID on:

- Factors that contribute to the development and/or maintenance of challenging behaviour?
- The process of assessing the function of challenging behaviour and the context in which it occurs?
- Interventions for challenging behaviour?
- Health professionals’ approaches to challenging behaviour?
- The use of medication for challenging behaviour?

## 2 METHOD

The results presented in this paper were obtained as part of a larger project in which a Dutch national multidisciplinary guideline for adults with ID and challenging behaviour is developed. This project is funded by the Dutch Ministry of Health, Welfare and Sport. As part of the guideline development, semi‐structured interviews were conducted with adults with ID and challenging behaviour or a history of challenging behaviour.

### 2.1 Selection and recruitment

Participants were selected on the basis of purposive sampling and had to fulfil the following criteria: be an adult (18+) with a mild or moderate intellectual disability and challenging behaviour or a history of challenging behaviour, and possess adequate verbal skills to communicate with the interviewer. Proxy interviews with family members or close associates were sought for adults with severe and profound ID or for those who were unable to communicate. Although the validity of the use of proxy interviews is debated, it has been stated that “a well‐informed guess may be preferable to no information in cases where the person is unable to communicate her/his own views” (Stancliffe, 1999). Since it is known that the prevalence of challenging behaviour increases with the level of disability (Poppes, van der Putten, & Vlaskamp, 2010), it was considered important to include proxy interviews in this study. Potential participants (both people with ID and proxies) were selected by members of the Guideline Development Group, made up of experienced professionals working in the care for people with ID and challenging behaviour. They acted as “gatekeepers” and established the first contact between the potential participant and the research team.

### 2.2 Data collection

The interviews were conducted using a semi‐structured topic list developed with members of the Guideline Development Group and two researchers experienced in interviewing people with intellectual disabilities. Topics were related to participants’ experiences with challenging behaviour, ways to reduce challenging behaviour, functional assessment, behavioural treatment and medication.

Interviews were conducted by one researcher and took place at a time and place that was convenient for the interviewee. Seventeen of the twenty interviews were conducted face‐to‐face at the residence of the participants to ensure their comfort and confidentiality; three of the interviews were conducted via telephone (all three were proxy interviews). The interviews lasted between 20 and 75 min and were audio‐taped, with the permission of the interviewees. Informed consent was obtained prior to commencing each interview, and afterwards, people with ID (not the proxies) received a voucher of €10 for giving their time.

### 2.3 Ethical considerations

The study design was sent to the Medical Ethical Committee of the Erasmus University Medical Center Rotterdam, the Netherlands, and judged as not being subject to the Medical Research Involving Human Subjects Act. Therefore, no formal medical ethics review was required. People with intellectual disabilities, after being selected by members of the Guideline Development Group, were first approached by either a family member or a trusted staff member to establish interest in the study. If the person with intellectual disability indicated that he or she would like to be interviewed, a member of the research team contacted the person and once more explained the interview proceedings and an appointment was scheduled. When preferred by the person with ID, a support person of their choice could be present during the interview. Prior to the interview, the interviewer explained the purpose of the interview and the principle of anonymity to the interviewees. Interviewees were assured that they could end the interview at any time and that they could refuse to answer any questions that made them feel uncomfortable. It was also explained that the information obtained from the interview was for research purposes while respecting their confidentiality. After these steps, informed consent was obtained.

### 2.4 Analysis

Interviews were recorded and subsequently transcribed verbatim. The qualitative data analysis software package ATLAS.ti 8 (ATLAS.ti Scientific Software Development GmbH) was used to organize the data. Analysis of the data was mostly deductive in nature, and data were coded to correspond to the research questions. Subsequently, an inductive approach was used to develop some additional codes. Coded data were linked and unified into themes. Initially, two researchers independently analysed three transcripts and compared the results with each other. Disagreements were resolved via discussion. After a sufficient level of agreement over the codes and themes was established, the remaining transcripts were coded by one researcher. Five themes emerged from the data; views and preferences (a) on factors that contribute to the development and/or maintenance of challenging behaviour, (b) regarding diagnostics, (c) on interventions, (d) concerning health professionals’ approaches and (e) on the use of medication for challenging behaviour. Initial analysis (not shown) indicated that responses given by people with ID were similar to those by proxies. Hence, it was decided to report the results combined for both groups. The results are narratively reported by theme, and quotations are chosen to illustrate the themes.

## 3 RESULTS

### 3.1 Characteristics of the sample

Thirteen adults with mild to moderate ID were interviewed (eight men and five women). Seven proxy interviews were conducted with relatives of adults with moderate, severe and profound ID (the adults with intellectual disability concerned were five men and two women). Of the people with intellectual disability that were interviewed, either directly or through a proxy, 12 had a mild ID, two had a moderate ID, and six had a severe/profound ID. Most of the persons with intellectual disability had multiple challenging behaviours: self‐injury/self‐harm (*n* = 9), aggression towards objects/destruction (*n* = 9), substance abuse (*n* = 7), aggression towards others (*n* = 6), oppositional behaviour (*n* = 6), anxiety (*n* = 5), inappropriate behaviour (*n* = 4), (thoughts of) suicide attempt (*n* = 4), wandering (*n* = 3), worrying (*n* = 3), withdrawal (*n* = 3), enuresis (*n* = 1), mood swings (*n* = 1) and verbal aggression (*n* = 1). The present authors did not explicitly ask for comorbid psychiatric diagnoses, but a number of diagnoses were mentioned (not exhaustive): (symptoms of) autistic spectrum disorders (*n* = 8), (symptoms of) schizophrenia or other psychotic disorders (*n* = 4), mood disorders (*n* = 2), anxiety disorders (*n* = 1) and gender identity disorders (*n* = 1).

### 3.2 Factors that contribute to the development and/or maintenance of challenging behaviour

Most interviewees attributed challenging behaviour at least partly to a lack of structure and clarity in their daily life. They were often unsure what staff members or others were expecting of them. This made them restless and afraid, and could potentially trigger challenging behaviour. This may also be partly connected to the (symptoms of) autism spectrum disorders that eight of the persons with intellectual disability were reporting. The interviewees mentioned that more clarity and structure from staff and others would help prevent challenging behaviour.

Other interviewees commented that challenging behaviour often resulted from staff or others placing too much demand on them. The persons with intellectual disability felt unable to live up to these expectations and, as a result, presented with challenging behaviour:

You are a smooth talker and so.. so people think you can handle everything, but that is not the case at all. So you can easily ask too much from me. And if they ask a lot of you, it will go wrong once. At a certain point, it will pile up and eventually you will fall over. (female with history of self‐injury/self‐harm, suicide attempt and aggression towards others)

Other frequently mentioned factors that are related to the development and/or maintenance of challenging behaviour are the inability to properly cope with emotions, feelings of loneliness and frustration when someone feels misunderstood or ignored, the loss of a loved one, an unsafe home situation or physical discomfort. According to the interviewees, clear rules, consistency, a limited number of choice options and being given time to get used to changes can contribute to the prevention of challenging behaviour.

### 3.3 Assessing the function of challenging behaviour and the context in which it occurs

The majority of people with ID did not have a strong opinion on the assessment process. Some did not remember the process while others mentioned they did not mind undergoing tests or observations. A small minority either explicitly liked the procedures—as it was interesting—or explicitly disliked it—because tests were too long, too difficult or people experienced pressure to achieve well. The interviewees perceived the results of the assessment process and tests in different ways. The most memorable for people was the moment they received a psychiatric diagnosis or the diagnosis of an intellectual disability. Some people who were diagnosed with a psychiatric disorder seemed to have difficulty with processing and accepting their diagnosis; they experienced the outcomes as confronting or did not agree with the outcomes. Others were glad that a diagnosis had been established, because it gave them clarity:

At first I found it very difficult. But I like to know it, because now, now I can anticipate on it. (female with history of aggression towards objects/destruction, withdrawal, self‐injury/self‐harm, suicide attempts)

### 3.4 Interventions for challenging behaviour

People with ID prefer to be informed about interventions. They like to give their opinion on possible interventions and want to be involved in the final decisions concerning the application of interventions. In the interviews, people with ID also gave their opinion about different types of interventions. Several interviewees were positive about doing sports or other physical activities to distract themselves or let off steam:

I just like to sport, that also provides some distraction (female, with history of wandering)

Other interventions that persons with ID considered useful are Eye Movement Desensitization and Reprocessing (EMDR) (*n* = 3), group therapy regarding addiction (*n* = 2) and assertiveness courses (*n* = 2). The use of seclusion and other restrictive measures was deemed controversial. One person felt that seclusion was aversive at all times, and another mentioned that she could understand the use of this measure afterwards.

It really was the best solution at that moment. At that moment I did not have that.. that thought. But in hindsight it really was the best solution, yes definitely. (female with history of aggression towards objects/destruction, withdrawal, self‐injury/self‐harm, suicide attempts)

Interviewees also mentioned positive experiences with psychoeducation; this helped them gain insight in their own behaviour and provided them with methods to cope with triggers for challenging behaviour in a more constructive manner. Similarly, people were positive about education given to caregivers. After a functional assessment developed a clear picture of the behaviour, the person, and the context, caregivers were informed about the best methods to support the person and to understand their actions better, for instance, by creating an early detection plan together, with the person and/or family, caregiver and psychologist. An early detection plan describes the signs of an emerging crisis in different phases and provides options for the person with intellectual disability or caregiver to prevent an escalation resulting in challenging behaviour. With such a plan, both persons with ID and caregivers could understand the progression of escalation of behaviour and were given tools to defuse a situation in an early stage.

### 3.5 Health professionals’ approach

As staff attitudes, approaches and support styles can play an important role in the prevention or reduction of challenging behaviour, interviewees were asked how they can be helped at the time they show challenging behaviour. Many interviewees found this a difficult question to answer. However, it became clear that in general, people with ID consider it detrimental if staff gets angry when people present with challenging behaviour. According to the interviewees, this leads to escalation of the situation. Often, people with ID find it pleasant to be left alone in the heat of the moment of the challenging behaviour and talk about it later when things have calmed down. Related to this, people with ID find it helpful if staff would sense and see what they need at such a moment. Sometimes, this information is explicitly included in the person's early detection plan, for example what staff should (not) say or do in certain circumstances. Finally, it became clear that people with ID find it important to get on well with the staff who treats and supports them, also in case of challenging behaviour:

Yes, in [previous residence], I had psychomotor therapy. But there I had two lessons. I’ve done that, and I did not have a connection with that woman, so I stopped. So I do need to click with the person, otherwise it’s of no use to me. (male, with history of substance abuse, self‐injury/self‐harm, worrying, withdrawal)

### 3.6 The use of medication for challenging behaviour

Ten of the thirteen people with ID that were interviewed were prescribed medication for challenging behaviour, most often antipsychotic medication. Other medicines that were used were benzodiazepines, antidepressants and methadone. In most cases, the present authors were not able to determine whether the medication was prescribed to treat a diagnosed disorder or symptom or whether it was an off‐label prescription. Almost all of the people with intellectual disability who received medication had (some) knowledge about their medication use; they knew the reason for prescription and the frequency with which they used it. Many were able to give the name of the medication, and some were also able to provide information on the dosage.

Some things have already been discontinued. But lorazepam has been taken off now, and per Wednesday one Haldol will also be taken off in the evening, then I'll only have one left in the evening. (male with history of thoughts of suicide attempt, substance abuse, withdrawal and worrying)

Some interviewees were pleased with their current medication and noticed its beneficial effects. However, several others expressed a wish to reduce or discontinue their current medication, or described previous discontinuation processes of the medication. The reasons were that people felt that the medication was not beneficial, addictive, could have side effects and/or should not be used unless absolutely necessary.

I got three actual medicines for things that were wrong with me. But if at a certain moment you get pills to deal with the side‐effects of the side‐effect medicine of the side‐effect medicine, then I’m like; what on earth are we doing? (male with (a history of) verbal aggression, aggression towards objects/destruction, aggression towards others, substance abuse and worrying)

Seven interviewees indicated that they were discussing their medication with their physician and that decisions to reduce or discontinue medication were taken in consultation with their physician. One of the interviewed persons with intellectual disability decided not to consult the physician and stop on his own. Those persons who were content with their current medication, mentioned that should they want to stop medication in the future, they knew that this could be discussed with their physician.

## 4 DISCUSSION

This study showed that people with ID and (a history of) challenging behaviour have clear views and preferences on the factors related to challenging behaviour, assessment, non‐pharmacological and pharmacological interventions and health professionals’ approach. These views and preferences are not always in accordance with the current procedures and treatments for challenging behaviour.

Many interviewees considered challenging behaviour to be, at least partly, a result from a lack of structure and clarity in their daily life and/or from staff or others placing too much demand on them. The inability to properly cope with emotions was also frequently mentioned by people with ID. This offers a starting point for developing an intervention and suggests that investing in a better understanding of peoples’ capabilities by staff and others—both in terms of their intellectual and adaptive skills—as well as offering counselling or training to better cope with emotions, may help reduce the occurrence of challenging behaviour in people with ID. The involvement of a person with intellectual disability in discussions regarding an intervention is important in this regard and may contribute to a higher success rate.

People with ID did not seem to remember much of the assessments undertaken with regard to the presence of challenging behaviour. However, when a psychiatric disorder was diagnosed, this left an impression. Some interviewees seemed to have difficulty with processing and accepting this diagnosis. As noted by Roy et al. (Roy, Roy, & Clarke, 2016), the emotional impact of a diagnosis can be substantial. Therefore, it is important that psychiatric diagnoses that may contribute to challenging behaviour are explained thoroughly, alongside possible treatment options, at a pace adapted to the individual person.

In line with earlier research (Griffith et al., 2013), the present authors found that people with ID have a strong interest in interventions for challenging behaviour; they like to give their opinion and want to be involved in the final decisions concerning the application of interventions. This is in line with previous studies that underscore the importance of participation by the person and other relevant stakeholders (Gore et al., 2013). In addition to previous studies that reported on the views of people with ID on specific therapies for different challenging behaviours (Hassiotis et al., 2013; Hays, Murphy, Langdon, Rose, & Reed, 2007; Pert et al., 2013; Westerhof, Beernink, & Sools, 2016), the present authors were able to help reduce the identified lack of information on experiences of specific psychological interventions (Griffith et al., 2013) by collecting positive attitudes on EMDR, group therapy regarding addiction and assertiveness courses. However, most interviewees were most enthusiastic about doing sports or other physical activities, perhaps because these are more “visible” and fun.

Persons who had experienced seclusion or restraint had strong views on the application of these interventions. Although none of the interviewees liked the use of seclusion and other restrictive measures, different opinions were held on the validity of their use; one person stated that physical restraint was always detrimental, but another interviewee agreed in hindsight that seclusion was the best option at that moment. It was felt that seclusion or restraint should not be applied too easily, but in some well‐specified situations the use of restraint could help to relax an agitated person. This is in line with the general attitude in the Netherlands, in which the use of restrictive measures is considered undesirable, and should only ever be used as a last resort. The baseline of the new Dutch Care and Coercion Act (in Dutch: Wet zorg en dwang), that will come into force in January 2020, is that coercive measures do not belong in the care for the disabled. What complicates the situation around restrictive measures further is that persons with ID do not always understand the reasons for the application of restrictive measures (MacDonald, McGill, & Deveau, 2011), while more understanding has been shown to help overcome negative feelings towards the intervention (Hawkins, Allen, & Jenkins, 2005). Hence, if seclusion or other physical interventions are used, it is important to clearly communicate and explain the reason for this to the person concerned.

Compared with psychological interventions for challenging behaviour, our interviewees seemed to be more aware and have more knowledge on pharmacological interventions. Almost all interviewees who received medication knew the reason for prescription, the frequency with which they used it, and many were also able to give the name and dosage of the medication. This is in contrast with a previous study from the UK (Hall & Deb, 2008), in which only a minority of people with ID was able to provide this information. The high level of awareness and knowledge on medication use was also reflected in strong preferences among interviewees regarding the discontinuation of medication. Several interviewees expressed a wish to reduce or discontinue their current medication. Much is still unknown about the discontinuation of medication in people with ID, especially where off‐label prescribed psychotropic medication is concerned. Yet in view of the importance of incorporating the experiences of people with ID in research and policy, this should be a key research priority and the subject should be structurally discussed with people with ID and their relatives.

Finally, the present authors found that many people with ID find it difficult to say what approach health professionals should take in case of challenging behaviour. In general, calmness of staff seems to be appreciated. While there is much attention in the literature on characteristics of care staff which are deemed important by people with ID in general, such as trustworthiness, engagement, acceptance, empathy and honesty (Frielink & Embregts, 2013), there is still a lack of knowledge on these issues in case of challenging behaviour.

In view of the prominence of challenging behaviour in the care for people with intellectual disabilities, its large negative impact and the importance of incorporating the experiences of people with ID in research and policy, this study identified the views and preferences of people with ID on various aspects of challenging behaviour and related care processes in the Netherlands. It was shown that people with ID consider a lack of structure in their daily life and staff placing too much demand on them, as important factors in the development of challenging behaviour. They have no strong views on the assessment process, but would like to be involved in decisions concerning interventions. Moreover, they seem to have a preference for some interventions and a strong desire to discontinue medication to the extent possible. These views and preferences are not always in accordance with the current procedures and treatments for challenging behaviour and should be included in future care processes and research.

### 4.1 Strengths and weaknesses

Several limitations of the study bear mentioning. First, the sample size was relatively small and prevents generalizing the findings of our study. Secondly, seven of the twenty interviews were conducted with proxies. While there is no conclusive evidence on the level of agreement between proxies and self‐reports by people with ID (Verdugo, Schalock, Keith, & Stancliffe, 2005), there is a strong preference to interview people with ID themselves whenever possible and only use proxies when absolutely necessary, for example due to significant communication limitations (van Asselt‐Goverts, Embregts, Hendriks, Wegman, & Teunisse, 2015). As all of our proxy interviews were conducted with family members or close associates of people with severe and profound ID and/or who were unable to communicate, and their expressed views and preferences were similar to those of the people with ID, the present authors believe the use of proxies was justified in our study.

Naturally, our study does not present a complete picture of the views and experiences of all people with ID on challenging behaviour, nor does it claim to do so. Yet considering the few studies currently available, and the dominance of UK‐based research in the literature, the findings of this study are a significant contribution to the knowledge base on the views and experiences of people with ID on challenging behaviour.

## CONFLICT OF INTEREST

None of the authors has any potential conflict of interest related to this manuscript.
